# Infantile Neuroaxonal Dystrophy: Diagnosis and Possible Treatments

**DOI:** 10.3389/fgene.2018.00597

**Published:** 2018-12-10

**Authors:** Patricia L. Babin, Sudheendra N. R. Rao, Anita Chacko, Fidelia B. Alvina, Anil Panwala, Leena Panwala, Danielle C. Fumagalli

**Affiliations:** ^1^Rare Genomics Institute, Downey, CA, United States; ^2^INADcure Foundation, Fairfield, NJ, United States

**Keywords:** infantile neuroaxonal dystrophy, rare disease, exome sequencing, enzyme replacement therapy, vector gene therapy, CRISPR/Cas9

## Abstract

Infantile Neuroaxonal Dystrophy (INAD) is a rare neurodegenerative disease that often cuts short the life span of a child to 10 years. With a typical onset at 6 months of age, INAD is characterized by regression of acquired motor skills, delayed motor coordination and eventual loss of voluntary muscle control. Biallelic mutations in the PLA2G6 gene have been identified as the most frequent cause of INAD. We highlight the salient features of INAD molecular pathology and the progress made in molecular diagnostics. We reiterate that enhanced molecular diagnostic methodologies such as targeted gene panel testing, exome sequencing, and whole genome sequencing can help ascertain a molecular diagnosis. We describe how the defective catalytic activity of the PLA2G6 gene could be potentially overcome by enzyme replacement or gene correction, giving examples and challenges specific to INAD. This is expected to encourage steps toward developing and testing emerging therapies that might alleviate INAD progression and help realize objectives of patient formed organizations such as the INADcure Foundation.

## Introduction

Infantile neuroaxonal dystrophy (INAD) is an autosomal recessive rare neurodegenerative disease of unknown frequency. The onset of symptoms generally occurs between 6 months and 3 years of age. Prior to that time, infants develop normally. The first symptom of INAD may be the slowing of the attainment of normal developmental milestones or regression in developmental milestones (Ramanadham et al., 2015). Trunk hypotonia, strabismus, and nystagmus are early symptoms of the disease (Gregory et al., 2017). The progression of the disease is rapid and as it progresses, more acquired skills are lost. Muscles soon become hypotonic and later become spastic (Levi and Finazzi, 2014). Eventually, all voluntary muscle control is lost. Muscle weakness can also lead to difficulties in feeding and breathing. In addition to nystagmus, some children experience vision loss. Cognitive functions are gradually lost and dementia develops. The life span is generally 5 to 10 years (Gregory et al., 1993; Jardim et al., 2004; Macauley and Sands, 2009).

## Molecular Pathology

Infantile Neuroaxonal Dystrophy belongs to a family of neurodegenerative disorders that includes atypical late-onset neuroaxonal dystrophy (ANAD) and dystonia Parkinsonism complex (DPC). Most cases of INAD are associated with homozygous or compound heterozygous mutations in the *PLA2G6* gene that affect the catalytic activity of its protein product (Engel et al., 2010). The *PLA2G6* gene encodes a group via calcium-independent phospholipase A2 protein (PLA2G6 or iPLA2β, ∼85/88 kDa) with a lipase and seven ankyrin repeat-containing domains (Tang et al., 1997). PLA2G6 hydrolyzes the sn-2 acyl chain of phospholipids, generating free fatty acids and lysophospholipids.

Phospholipids in the inner membrane of the mitochondria are rich in unsaturated fatty acids in the sn-2 position, particularly cardiolipin (Seleznev et al., 2006). These unsaturated fatty acids are particularly vulnerable to the abundant reactive oxygen species produced by the mitochondria (Murphy, 2009) resulting in peroxidized phospholipids in the inner membrane of the mitochondria. PLA2G6 localizes to the mitochondria (Williams and Gottlieb, 2002; Liou et al., 2005) consistent with an increased demand for hydrolysis of the peroxidized fatty acids in the sn-2 position of phospholipids leading to remodeled phospholipids (Balsinde et al., 1995; Zhao et al., 2010). When PLA2G6 is defective, the mitochondria inner membrane integrity is damaged. PLA2G6 also localizes to the axon (Ong et al., 2005; Seleznev et al., 2006) indicating an increased localized demand for phospholipid remodeling there as well. The manifestation of such accumulation in the brain is unique to key brain areas, such as the basal ganglia which resulted in various names for the same underlying molecular pathogenesis involving PLAG26 (Mehnaaz, 2016; Nassif et al., 2016).

**FIGURE 1:**
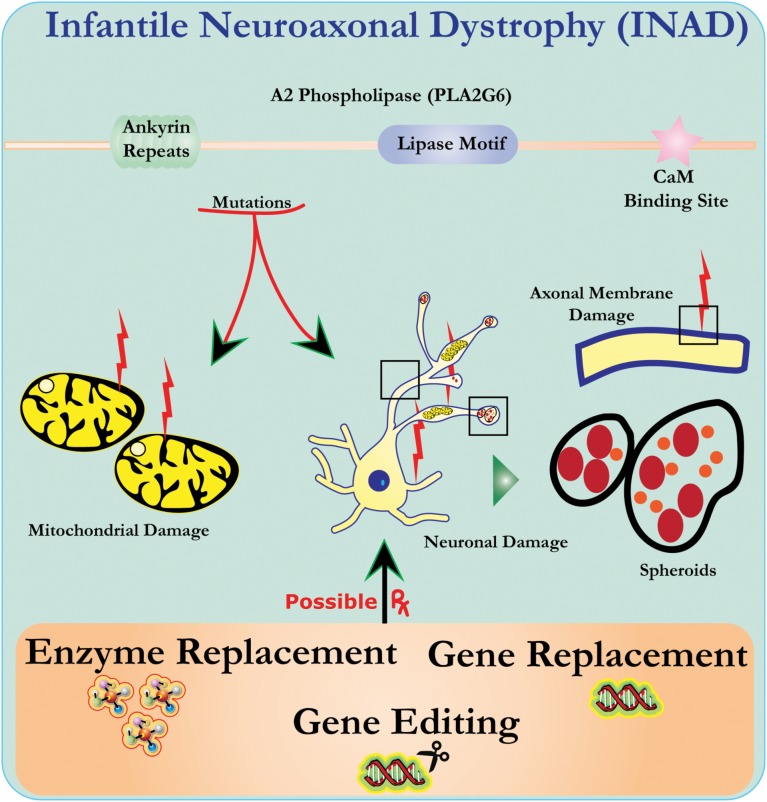
Infantile neuroaxonal dystrophy (INAD) is a neurodegenerative disorder related to mutations in the PLA2G6 gene. Various mutations of PLA2G6 lead to dysfunctional A2 phospholipase that leads to mitochondrial and axonal membrane defects. These defects cause neuronal damage visualized as axonal swellings and accumulation of pre-synaptic spheroids. Enzyme replacement to restore functions, gene replacement or editing to correct the defective PLA2G6 are proposed therapeutic strategies.

Ultrastructure analysis of neurons in *PLA2G6* knockout mice is consistent with this molecular pathology. Mitochondria with branching and tubular cristae, mitochondria with degenerated cristae, axons with cytoskeleton collapse, and partial membrane loss at axon terminals have been observed (Beck et al., 2011). At a microscopic level, these features appear as axonal swellings and spheroid bodies in pre-synaptic terminals (Figure 1) in the central or peripheral nervous system.

## Molecular Diagnostics and Rare Disease Patient Empowerment

Apart from specific clinical, electrophysiological, and imaging features, prior to the availability of next generation sequencing, skin biopsies showing axonal swellings and spheroid bodies in pre-synaptic terminals in the central or peripheral nervous system were the diagnostic criteria for the confirmation of INAD (Gregory et al., 2017; Iodice et al., 2017). Often, multiple biopsies were required to confirm the diagnosis. Families generally waited many years for a diagnosis. With the decreasing cost of gene and genome sequencing, availability of targeted gene panel testing with diagnostic labs for undiagnosed neurological diseases, and the increasing awareness of physicians of the availability of genetic diagnostics, families are receiving diagnosis more rapidly; sometimes within a year of the first symptom appearing. The children in these families are still young and the families are motivated to partner with scientists to find a treatment for their children’s illness. In order to fund the research process, a group of parents of INAD patients formed the INADcure Foundation. The foundation has raised substantial funds for research and is partnering with Rare Genomics Institute to guide them in the awarding of research grants. Interestingly, a 2016 genetic analysis of 22 Indian families with INAD, ANAD, and DPC found that 10/22 families (45.45%) lacked mutations in the *PLA2G6* gene coding region (Kapoor et al., 2016). Failure to identify deleterious mutations in the coding region of *PLA2G6* highlights that future molecular diagnostic efforts would require whole gene sequencing to identify mutations in the intronic and regulatory regions of the *PLA2G6* gene in INAD affected patients. Furthermore INAD cases where *PLA2G6* associated mutations are not observed suggests that the cause of the disease could be due to mutation in genes other than *PLA2G*, which needs to be explored.

## Possible Therapies

Most of the therapies that are considered for a rare disease like INAD involving a defective enzyme are enzyme replacement, gene replacement or gene correction. When an enzyme deficiency is caused by a recessive genetic defect, it is assumed that enzyme replacement or supplementation may correct the problem (Smith et al., 2012; Yu-Wai-Man, 2016). However, experiments to prove that therapies providing the correct gene or enzyme will rescue the INAD phenotype are yet to be performed and tested.

### Enzyme Replacement Therapy (ERT)

Since the brain is the primary organ affected in INAD, enzyme replacement therapy for INAD would most likely require infusion of the enzyme into the brain. In 2017, Biomarin received FDA approval for tripeptidyl peptidase 1 (cerliponase alfa) as a treatment for the underlying cause of Batten disease, the deficiency of tripeptidyl peptidase (TPP1) a lysosomal enzyme (U.S. Food Drug Administration, 2017). Tripeptidyl peptidase 1 (cerliponase alfa; ∼59 kDa) is the first ERT to be directly administered into the cerebrospinal fluid (CSF) of the brain. Other ERT drugs which can be administered into the CSF are in clinical trials (Jardim et al., 2004; Macauley and Sands, 2009).

The efficacy of tripeptidyl peptidase 1 (cerliponase alfa) on the walking ability of Batten disease patients demonstrates that enzyme replacement therapy for diseases that affect the brain is theoretically possible. Targeting replacement enzyme into the mitochondria for INAD will be more challenging than lysosomal targeting which had already been successful by IV ERT for a long time as in the case of Gaucher disease. Hence, there are still many biological issues specific to INAD that need to be resolved:

• Does therapy for INAD need mechanisms that can possibly transport PLA2G6 into the cell?
• What mechanism can be used to possibly localize PLA2G6 to the mitochondria?
• Is that sufficient or will PLA2G6 also need to be localized to the axon and if so, how?
• Is there a special need to address the targeting of the peripheral nervous system?
• Can a partial PLA2G6 rescue its deficiency or is a whole protein required?
• What is an appropriate solvent for delivery of *PLA2G6 gene product into the CSF*?
• If the PLA2G6 enzyme is not 100% pure, what are its contaminants and are they safe?
• Will there be any generalized intravascular complications due to PLA2G6 enzyme infusion into the CNS? Does the infusion dose have any relevance for such complications?

An additional issue is that enzyme replacement therapy requires the initial placement of an intracerebroventricular catheter and frequent infusions. The placement of the catheter requires anesthesia and the infusions may require anesthesia depending on the patients’ cooperation. Addressing the concerns of pediatric neurologists and parents of children with INAD with regard to using anesthesia on INAD patients with appropriate information will require further effort.

### Gene Therapy/Gene Replacement

The human *PLA2G* gene is around 70 kb with 17 exons and 2 alternate exons. The longest protein-coding transcript, however, is just 3.3 kb and the protein coding sequence is just over 2.4 kb, which can easily be packaged into a viral vector cargo. We will first explore another lysosomal storage disorder in the CNS that have preclinical data to then be compared with INAD. The lack of preclinical data of ERT in INAD is a significant knowledge gap in the field, which is why it is crucial that we observe other preclinical models of CNS disorders that are undergoing gene therapy studies. Previously, a preclinical study in mice addressed gene therapy for the lysosomal storage disorder mucopolysaccharidoses type IIIA (MPS-IIIA) by packaging the correct version of the sulphamidase gene inside a viral vector (Sorrentino et al., 2013). The study took advantage of the growing understanding of the blood brain barrier (BBB) which actively regulates the transport of large molecules from blood into CNS by a process called transcytosis (Pardridge, 2005b; Sorrentino and Fraldi, 2016).

Transcytosis involves endocytosis of ligands on the luminal side, mediated by ligand-specific receptors (e.g., insulin receptor, transferrin receptor, and low-density-lipoprotein receptor, etc.,) enriched on the capillary endothelium (Pardridge, 2005a). This is be followed by movement of endocytosed cargo through the endothelium cytoplasm and finally exocytosis at the abluminal (brain) side, thus effectively delivering the cargo across the BBB (Pardridge, 2002). The MPS-IIIA preclinical study manufactured a chimeric sulphamidase protein that contained a BBB-binding domain from apolipoprotein B to facilitate uptake by the endothelium and also a signal peptide from iduronate-2-sulfatase to aid an efficient exocytosis toward the abluminal side of the BBB (Sorrentino et al., 2013). A viral vector cargo encoding a chimeric sulphamidase was then loaded onto an adeno-associated virus (AAV) serotype 2/8 targeting the liver (Sorrentino and Fraldi, 2016). Thus the liver served as an internal factory that provided a constant supply of chimeric-sulphamidase, which resulted in a 10–15% increase in brain sulphamidase activity even 7 months after liver gene therapy. This increase in brain sulphamidase activity levels led to quantifiable improvement in brain pathology and behavior outcomes in the mouse model of MPS-IIIA (Sorrentino et al., 2013).

If a similar study is conducted for INAD, it would answer several critical questions about enzyme replacement for INAD therapy. Alternatively, intra-vascular or intra-CSF administration of AAV9 based gene therapy products can directly target CNS (Bey et al., 2017; Roca et al., 2017). However, the multiple mutations that INAD patients have on their PLAG26 gene provides unique challenges to gene therapy. Though the size of the PLA2G6 gene of 2–3 kb should not pose a problem for its insertion into a viral vector, however, the regulatory complications from correcting PLA2G6 are difficult to predict. Regulatory complications become uncontrollable especially if the gene therapy cannot be localized properly within the target tissues. Enzyme replacement therapy may also be a problem if some mutant products turn out to be dominant negative to the wild-type PLAG26.

Most of the challenges of possible therapies for INAD comes from the fact that it is an ultra-rare disease. However, the fact that INAD has a known genetic etiology provides avenues for possible therapies. Moreover, any successful therapy for INAD will receive orphan drug status and all the protection it gets because INAD is a rare disease. Thus despite all the aforementioned challenges, the orphan drug status provides a strong incentive for rare disease researchers and the biotechnology industry, not to mention when the genetic cause is already known.

### Gene/Base Editing

As of 2017, at least 277 missense mutations have been observed in the human *PLA2G6* gene (Lek et al., 2016). Only a small proportion of *PLA2G6* mutations include frame shifts, indels, nonsense mutations and mutations in the splice sites (Morgan et al., 2006). Thus correcting the gene in the target cell population or correcting the mutated DNA bases is an attractive therapeutic avenue. Over the years, several tools have been developed for gene editing, and CRISPR/Cas9-based genome editing is being hailed as a ground breaking technology with a clinical trial planned in 2018. These technologies utilize a DNA binding protein that can also cleave the strand in a specified manner to make space for the insertion of a new DNA sequence or correction of the deleterious DNA base (LaFountaine et al., 2015). CRISPR/Cas9 technology has already yielded astounding therapeutic results in several pre-clinical disease models (e.g., Duchene muscular dystrophy, liver metabolic diseases, etc.,) (Dai et al., 2016). Additionally, a growing understanding of human variation is posing newer challenges to gene therapy and driving innovation toward a true personalization of gene editing (Lessard et al., 2017; Scott and Zhang, 2017). New technologies such as vSLENDR, an AAV virus, and CRISPR/Cas9-mediated technology to replace defective genes in neurons and other nervous system cells are pushing gene editing technologies to new frontiers (Nishiyama et al., 2017).

## Conclusion

Infantile Neuroaxonal Dystrophy is a severe neurodegenerative disease with a certain morbidity and mortality. This rare disease offers an exciting opportunity to revalidate available modes of next generation therapy and generate newer ones. Emerging congruence on the clinical diagnostic criteria for INAD is expected to provide an impetus toward enhanced molecular diagnostics. This progress is expected to lead us to developing affordable therapies that would provide quantifiable improvement in the quality of life of INAD patients and retard or ameliorate the disease progression. We have highlighted some success stories in Batten’s disease and the mucopolysaccharidoses that provide us with the inspiration to ask the right questions to make INAD therapy a reality. In addition, growing viral and non-viral approaches for CRISPR/Cas9 based gene editing should also open newer therapeutic avenues for INAD.

## Author Contributions

PB and FA prepared preliminary draft. SR and AC edited and added more sections to the manuscript. DF, AP, and LP edited the manuscript.

## Conflict of Interest Statement

The authors declare that the research was conducted in the absence of any commercial or financial relationships that could be construed as a potential conflict of interest.
